# Physical activity and motivational readiness for physical activity behavior change in adults with non-communicable diseases in Germany: a trend analysis of two cross-sectional health surveys from the German GEDA study 2014/2015 and 2019/2020

**DOI:** 10.1186/s12889-025-21507-y

**Published:** 2025-02-06

**Authors:** Benjamin Wenz, Jonathan Graf, Gorden Sudeck, Wolfgang Geidl, Kristin Manz, Susanne Jordan, Andrea Teti, Lars Gabrys

**Affiliations:** 1 Institute of Gerontology, Faculty I, Vechta University, Vechta, Germany; 2 University of Applied Sciences for Sport and Management Potsdam, Potsdam, Germany; 3 Institute of Sport Science, University Tübingen, Tübingen, Germany; 4 Interfaculty Research Institute for Sport and Physical Activity, University Tübingen, Tübingen, Germany; 5 Department of Sport Science and Sport, Friedrich-Alexander-Universität Erlangen-Nürnberg, Erlangen, Germany; 6 Department for Epidemiology and Health Monitoring, Robert Koch Institute, Berlin, Germany

**Keywords:** Non-communicable disease, Physical activity, Exercise, Motivational readiness, Transtheoretical model

## Abstract

**Background:**

Physical activity (PA) is a cornerstone in maintaining a healthy lifestyle as well as in the prevention and rehabilitation of non-communicable diseases (NCD). First analysis of PA showed lower activity rates in adults with NCD compared to the general population. To improve health monitoring and to address World Health Organization (WHO) recommendations to systematically identify and track efforts to reduce inequalities in PA participation, trend analysis of PA in adults with NCD was performed for the period 2014/15 to 2019/20 for Germany. Furthermore, motivational readiness for PA behavior change was analysed based on the transtheoretical model (TTM).

**Methods:**

Based on two population-based cross-sectional health surveys (GEDA 2014/15-EHIS and GEDA 2019/20-EHIS) for Germany (*N* = 46,724), the prevalence of meeting WHO PA guidelines was analysed for adults with certain NCD compared to the general population. PA was assessed by self-report via the European Health Interview Survey– Physical Activity Questionnaire (EHIS-PAQ). Trend analyses and logistic regression models were performed to calculate disease specific Odds Ratios (OR) for fulfilment of PA recommendations. Motivational readiness for PA was assessed with the stages of change according to the TTM with data of GEDA 2014/15-EHIS.

**Results:**

Reporting any NCD is associated with lower fulfilment of health-enhancing aerobic PA in both surveys for almost all NCD, with lowest levels among adults reporting stroke, type 2 diabetes mellitus, chronic obstructive pulmonary disease, obesity and coronary heart disease. Sufficient muscle-strengthening was higher in adults with musculoskeletal diseases like osteoarthritis, lower back pain and neck pain compared to adults without these diseases. The prevalence of meeting WHO PA recommendations among adults with NCD remains at a low level. Sufficient health-enhancing aerobic PA tend to decrease in adults with NCD from 2014/15 to 2019/20, while sufficient muscle-strengthening increased in the same period. Motivational readiness for PA is lower for most adults with NCD, compared to the general adult population.

**Conclusion:**

Lower rates of WHO PA recommendation fulfilment is recognized for most NCD groups compared to the population without NCD for both surveys, but the proportion of adults with NCD who meet the WHO PA recommendations differ widely between NCD groups. A positive trend from 2014/15 to 2019/20 can only be seen for adults with osteoarthritis. Based on our findings the implementation of PA promotion particular with regards to motivational readiness and disease specific PA measures is strongly recommended to improve prevention and ambulatory health care for adults with NCD.

## Background

Chronic non-communicable diseases (NCD) such as cardiovascular diseases (CVD), chronic respiratory diseases (CRD), diabetes mellitus (DM) and cancer are of great importance to public health and for healthcare systems [1]. Cardiovascular diseases, cancer, and cerebrovascular diseases have accounted for more than 70% of all deaths in Germany for the last three decades and will remain the major driver of mortality in Germany over the next decades [2]. NCD are characterised by long duration and slow progression and impose a high burden, both financially and structurally, on the health care system [3]. Physical activity (PA) is a cornerstone in maintaining health for adults with NCD. PA is not only effective in reducing the risk for the development of numerous NCD but also in the treatment of those diseases like diabetes mellitus type 2 (DMT2), coronary heart disease (CHD), different types of cancer, musculoskeletal and lung diseases as well as mental illnesses (e.g. depression) and is an important component in maintaining health [4, 5]. Besides medication and diet, PA is a strongly recommended in international and national treatment guidelines for many NCD [6–11].

At the same time, a majority of the German adult population is not sufficiently physically active and does not meet PA guidelines. Therefore, promoting PA is an important preventive measure in health policy [12]. Looking at PA trends and insufficient PA between 2000 and 2022 on a global level, Germany has been one of the countries with a positive progress towards reducing the prevalence of insufficient PA among high-income Western countries in the last three decades [13]. Based on national health monitoring data (GEDA 2019/20-EHIS) it has been shown that health-enhancing aerobic PA in the general adult population increased only slightly, with more than 50% being insufficiently physically active [14, 15]. Moreover, several international studies and also data for Germany show lower levels of PA in chronically ill people compared to healthy individuals [16–21].

Although, World Health Organization (WHO) recommends to undertake NCD risk factor surveillance (including PA) on a national level and to strengthen reporting systems to enable health monitoring of trends within subpopulations like persons with NCD, a previous study by Sudeck et al. in 2021 was the first nationwide analysis of PA behavior in persons with NCD for Germany [22].

For a better understanding and to assess the potential for PA behavior change in different target groups, as for example adults with NCD, psychological theories like the transtheoretical model (TTM) for behavior change must be considered. The TTM, also known as the stages of change model was initially developed by Prochaska and DiClemente in the context of smoking cessation [23]. Later it has been applied to PA by Marcus et al. [24] and is widely used to assess readiness to adopt exercise and PA behaviors [25–26]. The TTM is based on the assumption that individuals go through different phases– precontemplation, contemplation, preparation, action, and maintenance– for changing behavior [23]. To date, there has been only little research on the stages of change in adults with NCD in Germany.

Against this background, the present paper focuses on (1) providing trend analysis of PA behavior in adults with the following nine NCD (coronary heart disease, stroke, type 2 DM, obesity, COPD, osteoarthritis, low back pain, neck pain and depressive symptoms) between 2014/15 and 2019/20 based on German national health monitoring data and (2) the investigation of the motivational readiness for PA behavior among adults reporting specific NCD. The concept of motivational readiness has been used to estimate the intention to change PA behavior, therefore we aim to investigate whether adults with NCD show different patterns of stages of change compared to adults without NCD. To sum up, our analysis can provide explanations for differences in activity levels among adults with NCD as well as important insights of psychosocial characteristics of this target group. Both analyses can contribute to better conceptualize tailored PA programmes in ambulatory health care and stationary medical rehabilitation for adults with NCD. Furthermore, the trend analysis can serve as a basis for a regular reporting system of PA in adults with NCD in Germany.

## Methods

### Study design and participants

The analyses are based on national health monitoring data. The German Health Update (Gesundheit in Deutschland aktuell, GEDA) is a nationwide cross-sectional health interview survey based on a random sample of adults with permanent residency in Germany conducted by the Robert Koch Institute (RKI) on behalf of the German Federal Ministry of Health (GEDA 2014/15-EHIS, *n* = 24,016 and GEDA 2019/20-EHIS, *n* = 22,708). GEDA is part of the ongoing health monitoring conducted by the RKI. GEDA data is based on self-reports of the participants. GEDA 2014/15-EHIS was either conducted as a paper or online questionnaire, [27–28] with a response rate of 26.9% [29]. GEDA 2019/20-EHIS was conducted as a telephone interview survey with a response rate of 21.6% [30]. The study design, objectives, and methods of GEDA 2014/15-EHIS and GEDA 2019/20-EHIS are described in detail elsewhere [29]. The study protocols were inspected and approved by the Federal Commissioner for Data Protection and Freedom of Information in Germany. Written informed consent was obtained from all participants. Participants were informed about study aims and content as well as privacy and data protection proceedings. Study participation was always voluntary. The Ethics Committee of the Charité– Universitätsmedizin Berlin, Germany assessed the ethics of GEDA 2019/20 [31].

### Study variables

#### Socio-demographic and anthropometric characteristics

Both surveys assessed socio-demographic variables like sex, age and educational level (low, middle, high) based on the parameters defined in the Comparative Analysis of Social Mobility in Industrial Nations (CASMIN) [32] as well as self-rated anthropometric variables like body weight in kg and height in cm. To prepare for further analysis age was grouped into four age categories (18–29, 30–44, 45–64, and ≥ 65 years). The body mass index (BMI) was calculated by using the following formula: BMI = body weight (kg)/(body height (m))^2^. BMI categorization was defined according to WHO’s definition as normal weight (< 25 kg/m^2^), overweight (25– <30 kg/m^2^), and obesity (≥ 30 kg/m^2^) [33].

#### Non-communicable diseases

GEDA questionnaires ask regularly for the prevalence of numerous NCD. Data on the 12-month prevalence of chronic diseases are based on responses to the following question: “This section deals with lasting diseases and chronic health problems. In the past 12 months, have you had any of the following diseases or health problems?” (possible answers: “yes” or “no”) [30]. For our analysis, cases with CHD, stroke, type 2 DM, obesity, COPD, osteoarthritis, low back pain, neck pain and depressive symptoms were selected for PA behavior analysis to compare with the respective cases not reporting this specific NCD.

#### Physical activity

Leisure-time PA behavior was assessed by self-report via the European Health Interview Survey– Physical Activity Questionnaire EHIS-PAQ [34] and the achievement of fulfilling the WHO recommendations for health-enhancing PA was used for analysis. EHIS-PAQ differentiates between aerobic and muscle-strengthening activities. Regarding the WHO PA recommendations, adults should fulfil at least 150 min of moderate to vigorous PA (MVPA) throughout the week and perform muscle-strengthening at least twice a week [35]. Participants in both surveys were asked about the amount of time they spent in health-enhancing aerobic PA for recreation and cycling for travelling from place to place per day as well as the number of days per week they engage in muscle-strengthening [34]. Based on the PA compendium of Ainsworth, time adults spent for walking was not included in the analysis because of not meeting the cut-off for moderate PA. Finally, the duration of PA for recreation and cycling for travelling from place to place per week was used to estimate whether 150 min moderate to vigorous physical activity (MVPA) was fulfilled or not [36]. This is in line with the development of the EHIS-PAQ instrument by Finger et al. (2015) and is used as the indicator for regular health monitoring [37]. Both surveys used the same questionnaire and definition for fulfilling WHO PA recommendations. For our analysis we used the following definitions for fulfilling WHO PA recommendations: (i) fulfilling 150 min of moderate aerobic PA, (ii) fulfilling a minimum of muscle-strengthening two times a week and (iii) fulfilling both recommendations.

#### Motivational readiness for physical activity

The analysis of motivation and barriers to PA realization is based on GEDA 2014/15-EHIS data. Motivational readiness was assessed with the internationally used and validated instrument (stages of change for PA) and adapted for the GEDA 2014/15-EHIS survey, which uses five stages of behavior change: *(i) precontemplation*,* (ii) contemplation*,* (iii) preparation*,* (iv) action and (v) maintenance* [38].

Motivational readiness for PA was asked as follows:

i.) “We have already asked you about the frequency and duration of physical and sporting activity in your leisure time in a typical week. For how many months have you been physically active or inactive in this way?” (“less than 6 months” or “6 months or more”).
ii.) “Do you plan to be physically active more often than before?” (“yes” or “no”).
iii.) In case of a positive answer to the second question, the following question was asked: “When do you plan to be physically active more often than before?” (“in the next 30 days” or “in the next 6 months”).

**Fig. 1:**
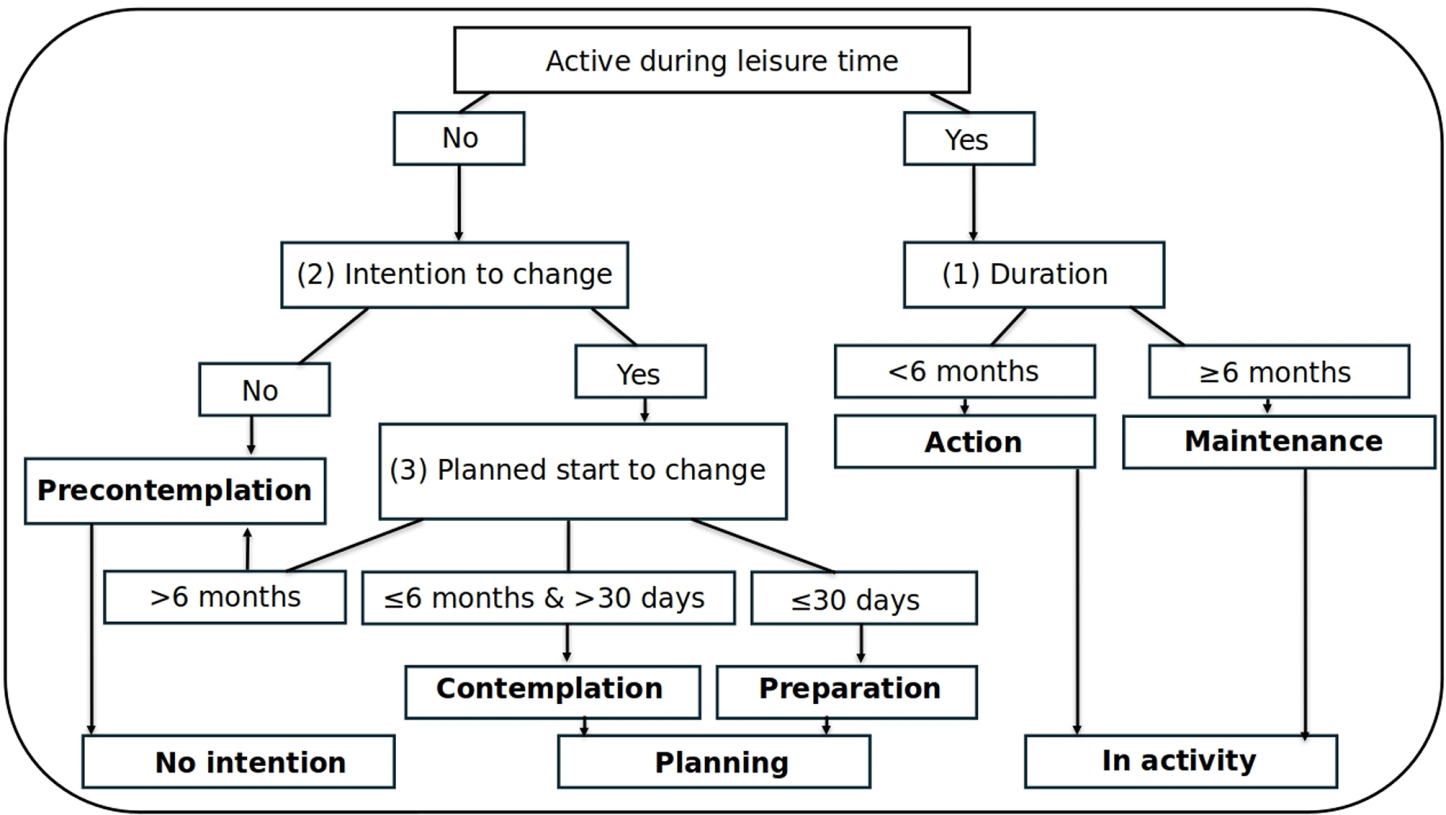
Algorithm to calculate the stages of change for physical activity, by Buchmann et al. 2023 [38]

For the analyses to calculate the stages of change for PA we used the algorithm developed by Buchmann et al. (2023) that is based on earlier work by Ronda et al. and is illustrated in Fig. 1 [38].

Stages of change for the implementation of PA are defined as follows:

*Precontemplation*: people have no intention to start being physically active (e.g. in the next six months).

*Contemplation*: people face their problem and seriously consider changes from inactivity to physical activity in the next six months. However, they have not yet made a commitment to act.

*Preparation*: combination of intentional and behavioral aspects. Individuals in this stage are willing to take action within the next 30 days and may have already taken some steps towards behavior change.

*Action*: people have been actively restructuring their behavior, their experiences or their environment for being physically active for about six months.

*Maintenance*: people maintain the changes they have achieved in terms of PA for more than six months and prevent possible relapse. The application of the learned skills and strategies now finally become routine. The duration of this stage approximately lasts between six months and five years.

### Statistical analyses

The trend analysis of the associations between PA and NCD was performed for the GEDA 2014/15-EHIS and GEDA 2019/20-EHIS health surveys. For both surveys’ identical definitions of NCD and PA which are described before were applied. As motivational readiness to PA was only available for GEDA 2014/15-EHIS, it is not part of the trend analysis.

Next to descriptive analysis of the study sample, adjusted multivariable logistic regression models were applied to calculate associations between NCD and fulfilment of WHO’s PA recommendations for both surveys as well as for trend analysis. A statistically significant difference between groups is assumed if the corresponding p-value is less than 0.05. All results are shown with a point estimate of the percentage of fulfilling the WHO’s recommendations with a 95% confidence interval (95% CI). The analyses focus on the fulfilment of the WHO recommendations for health-enhancing aerobic PA, muscle-strengthening and the combination of both recommendations. In the regression model the PA fulfilment of the WHO recommendation was the dependent variable. As exposure variables we used the following NCD groups (CHD, stroke, DM, obesity, COPD, osteoarthritis, low back pain, neck pain and depressive symptoms). Survey weights were applied to enable representative analyses for the German population [31]. The group comparison was made in relation to individuals not suffering from the respective NCD serving as the reference group. Results are presented as odds ratio (OR) and corresponding 95% CI. To account for potential confounders the results were adjusted for sex, age and educational level. For GEDA 2014/15-EHIS the motivational readiness for PA is shown as a percentage of five stages (i) precontemplation, (ii) contemplation, (iii) preparation, (iv) action, (v) maintenance for each of the nine different NCD. All the analyses were carried out with SAS 9.04.

## Results

The characteristics of the study participants in GEDA 2014/15-EHIS and GEDA 2019/20-EHIS are presented in Table 1. In GEDA 2019/20-EHIS the prevalence of low back pain (32.0%) and neck pain (23.4%) are the highest. The prevalence of stroke (1.8%) was the lowest among participants. The prevalence of CHD, stroke, DM, COPD and osteoarthritis was highest in the oldest age group (65 years and older) with except for obesity, low back pain, neck pain and depressive symptoms which was highest in the middle age group (45–64 years). NCD prevalences do not differ significantly between the two surveys GEDA 2014/15-EHIS and GEDA 2019/20-EHIS, except for low back pain and neck pain for which the prevalence has been lower in GEDA 2019/20-EHIS compared to GEDA 2014/15-EHIS.

**Table 1:** Distribution and characteristics for different NCD in two GEDA survey waves (2014/15-EHIS and 2019/20-EHIS), a nationwide study population of German adults (+ 18 years)

	GEDA 2014/15-EHIS				GEDA 2019/20-EHIS			
	Cases (%)	Female (%)	Age (%)	Education (%)	Cases (%)	Female (%)	Age (%)	Education (%)
			18–29 y	low			18–29	low
			30–44 y	middle			30–44	middle
			45–64 y	high			45–64	high
			65 + y				65+	
**General adult population**	24.016 (100)	51.1	16.9	31.3	22.708 (100)	51.1	15.5	29.6
			22.2	53.1			22.4	52.3
			36.4	15.5			34.9	18.0
			24.5				27.2	
**Coronary heart disease**	907 (4.2)	40.9	1.0	58.5	1.124 (4.6)	48.1	1.1	53.0
			1.8	32.4			2.3	35.8
			25.6	9.2			28.2	11.2
			71.6				68.4	
**Stroke**	249 (1.2)	51.3	0.4	64.2	384 (1.8)	48.6	0.4	51.9
			4.5	29.6			5.2	39.2
			23.8	6.1			35.5	8.8
			71.				58.9	
**Type 2 diabetes mellitus**	1.712 (7.7)	46.1	1.7	52.9	2.059 (8.9)	46.8	0.8	51.6
			5.0	38.5			7.9	38.2
			34.0	8.6			34.4	10.2
			59.2				56.9	
**Obesity**	4.049 (18.1)	50.6	8.7	41.5	3.875 (19.0)	50.6	7.7	41.5
			21.2	49.3			19.5	48.2
			41.7	9.1			42.7	10.3
			28.4				30.1	
**COPD**	1.230 (5.8)	51.2	5.5	50.1	1284 (6.1)	53.7	2.8	54.1
			10.7	41.5			11.7	37.5
			36.1	8.4			42.5	8.3
			47.6				43.0	
**Osteoarthritis**	3.897 (17.9)	62.0	0.6	49.1	4.384 (17.1)	64.5	1.0	44.7
			5.3	40.8			5.3	43.4
			40.6	10.2			39.2	11.8
			53.5				54.5	
**Low back pain**	9.040 (40.1)	54.2	11.9	37.0	7.175 (32.0)	53.9	8.8	39.3
			19.1	50.7			17.2	49.0
			39.3	12.3			40.1	11.8
			29.7				33.9	
**Neck pain**	7.976 (34.3)	61.3	12.3	32.7	5.266 (23.4)	63.4	8.5	36.5
			20.8	54.1			18.5	50.7
			41.6	13.3			41.7	12.9
			25.3				31.3	
**Depressive symptoms**	2.499 (11.3)	58.1	16.1	36.0	2.063 (11.8)	57.5	17.5	35.9
			22.6	52.8			22.6	54.3
			43.3	11.2			41.5	9.8
			18.0				18.4	

### Fulfilment of PA recommendations in adults with NCD

Table 2 shows the self-reported fulfilment and trend analysis of WHO PA recommendation for nine NCD in GEDA 2014/2015-EHIS and GEDA 2019/20-EHIS for the following dimensions: (i) health-enhancing aerobic activity (ii) muscle-strengthening and (iii) both recommendations combined.

In 2019/20 the fulfilment of health-enhancing aerobic PA was lowest for stroke (19.9% [95% CI 14.9–24.9%]), type 2 DM (28.9% [95% CI 25.9–31.9%]), COPD (30.0% [95% CI 26.0-33.9%]), obesity (33.1% [95% CI 30.9–35.3%]) and CHD (33.2% [95% CI 29.0-37.5%]). For adults reporting any of the analysed NCD the fulfilment of muscle-strengthening was lowest for stroke (23.6% [95% CI 17.5–29.8%]), type 2 DM (24.8% [95% CI 22.0-27.7%]), obesity (25.7% [95% CI 23.7–27.8%]), CHD (28.4% [95% CI 24.2–32.5%]) and COPD (31.0% [95% CI 27.0–35.0%]). In the general adult population, the fulfilment of health-enhancing aerobic PA (48.0% [95% CI 47.0–49.0%]) was considerably higher compared to muscle-strengthening (36.3% [95% CI 35.3–37.2%]). In general, adults reporting any of the nine analysed NCD were less physically active in each of the three dimensions compared to the general adult population. Trend analysis showed generally higher rates in the fulfilment of WHO PA recommendations in the general population in 2019/20 compared to 2014/15. In contrast, significantly lower rates of health-enhancing aerobic activity could be observed in adults with stroke and osteoarthritis in 2019/20 compared to 2014/15. For muscle-strengthening higher rates could be observed in adults with obesity, type 2 DM, COPD, low back pain, neck pain and depressive symptoms, in 2019/20 compared to 2014/15.

**Table 2:** Self-reported fulfilment of WHO physical activity recommendation (health-enhancing aerobic activity, muscle-strengthening and both recommendations combined) for nine non-communicable diseases in GEDA 2014/15-EHIS and GEDA 2019/20-EHIS

	Aerobic activity (≥ 150 min/week) GEDA 2014/15-EHIS and GEDA 2019/20-EHIS (without walking)			Muscle-strengthening ≥ 2 times/week			Both recommendations for aerobic activity and muscle-strengthening combined fulfilled		
	%	(95% CI)	*p*-trend	%	(95% CI)	*p*-trend	%	(95% CI)	*p*-trend
General adult population (reference population)									
GEDA 2014/15	45.3	44.5–46.0	**< 0.001**	29.4	28.7–30.1	**< 0.0001**	22.5	21.9–23.2	**< 0.0001**
GEDA 2019/20	48.0	47.0–49.0		36.3	35.3–37.2		26.3	25.4–27.1	
Non-communicable diseases (NCD)									
**Coronary Heart disease**									
GEDA 2014/15	38.2	34.1–42.3	0.100	26.7	23.0-30.5	0.568	19.7	16.2–23.3	0.063
GEDA 2019/20	33.2	29.0-37.5		28.4	24.2–32.5		15.2	11.6–18.5	
**Stroke**									
GEDA 2014/15	29.4	22.0-36.8	**0.029**	28.5	21.6–35.5	0.298	18.2	11.9–24.6	**0.002**
GEDA 2019/20	19.9	14.9–24.9		23.6	17.5–29.8		8.7	5.9–11.8	
**Type 2 diabetes mellitus**									
Geda 2014/15	32.7	29.9–35.4	0.070	21.0	18.7–23.3	**0.043**	14.0	12.0-16.1	0.298
Geda 2019/20	28.9	25.9–31.9		24.8	22.0-27.7		12.5	10.3–14.6	
**Obesity**									
GEDA 2014/15	31.7	29.9–33.4	0.328	19.6	18.2–21.1	**< 0.0001**	12.9	11.7–14.2	**0.024**
GEDA 2019/20	33.1	30.9–35.3		25.7	23.7–27.8		15.3	13.7–17.0	
**COPD**									
Geda 2014/15	30.0	26.8–33.2	0.983	25.4	22.4–28.4	**0.028**	16.5	13.9–19.2	0.808
GEDA 2019/20	30.0	26.0-33.9		31.0	27.0–35.0		16.0	12.8–19.2	
**Osteoarthritis**									
GEDA 2014/15	43.6	41.7–45.6	**< 0.0001**	31.7	29.9–33.5	0.084	22.8	21.2–24.5	0.265
GEDA 2019/20	36.9	34.8–39.1		34.1	32.0-26.2		20.1	18.3–21.9	
**Low back pain**									
GEDA 2014/15	41.4	40.0-42.6	0.882	29.5	28.4–30.7	**< 0.0001**	21.2	20.2–22.3	**0.039**
GEDA 2019/20	41.2	39.4–43.0		35.8	34.2–37.5		23.1	21.7–24.6	
**Neck pain**									
GEDA 2014/15	41.9	40.5–43.2	0.964	29.0	27.8–30.2	**< 0.0001**	20.7	19.6–21.8	0.0660
GEDA 2019/20	42.0	39.9–44.0		35.9	34.0-37.9		22.5	20.8–24.2	
**Depressive symptoms**									
GEDA 2014/15	36.9	34.4–39.2	0.271	25.5	23.4–27.6	**< 0.0001**	17.9	16.0-19.7	0.4505
GEDA 2019/20	34.7	31.6–37.8		33.5	30.4–36.7		19.1	16.5–21.7	

**Fig. 2:**
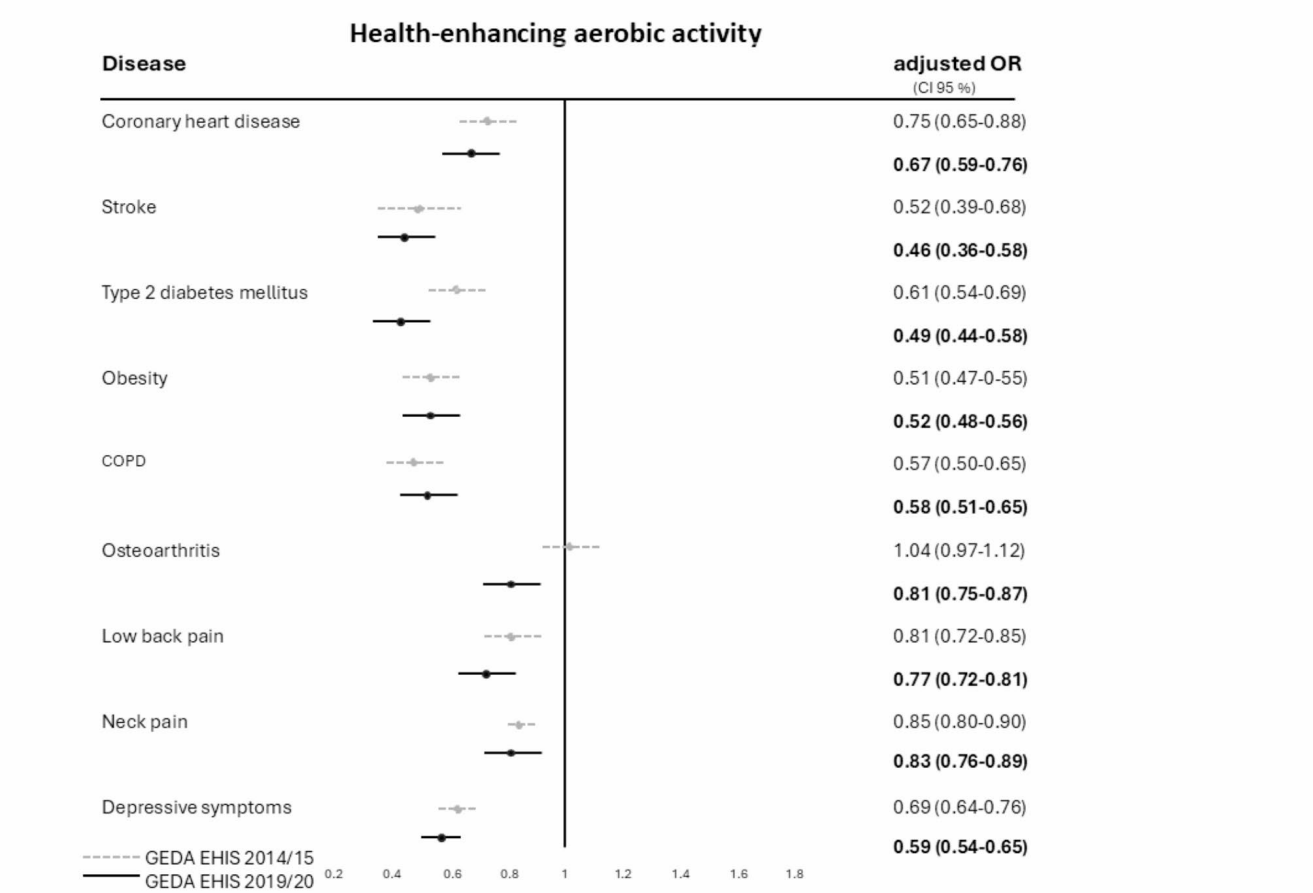
Forest plot for multivariate logistic regression with odds ratios (OR), adjusted for sex, age group and educational level and 95% confidence intervals (CI) for the fulfilment of recommendations for health-enhancing aerobic PA (at least 150 min. per week) for nine NCD in two national health surveys (GEDA 2014/15-EHIS and GEDA 2019/20-EHIS) in Germany. Reference category: certain NCD not reported

Figure 2 shows the analysis of adjusted logistic regression models (adjusted for sex, age group and education) for nine NCD in GEDA 2014/15-EHIS and GEDA 2019/20-EHIS compared to participants without this specific NCD. The lowest OR of fulfilling the WHO recommendations for health-enhancing aerobic PA with the strongest association was found for stroke (OR = 0.46 [95% CI 0.36–0.58%]), type 2 DM (OR = 0.49 [95% CI 0.49–0.58%]), obesity (OR = 0.52 [95% CI 0.48–0.56%]), COPD (OR = 0.58 [95% CI 0.51–0.65%]) and depressive symptoms (OR = 0.59 [95% CI 0.54–0.65%]). The fulfilment of health-enhancing aerobic PA in adults with NCD tend to be stable since 2014/2015 (compare GEDA EHIS-2014/15) and remains low compared to the general adult population (see Fig. 2).

**Fig. 3:**
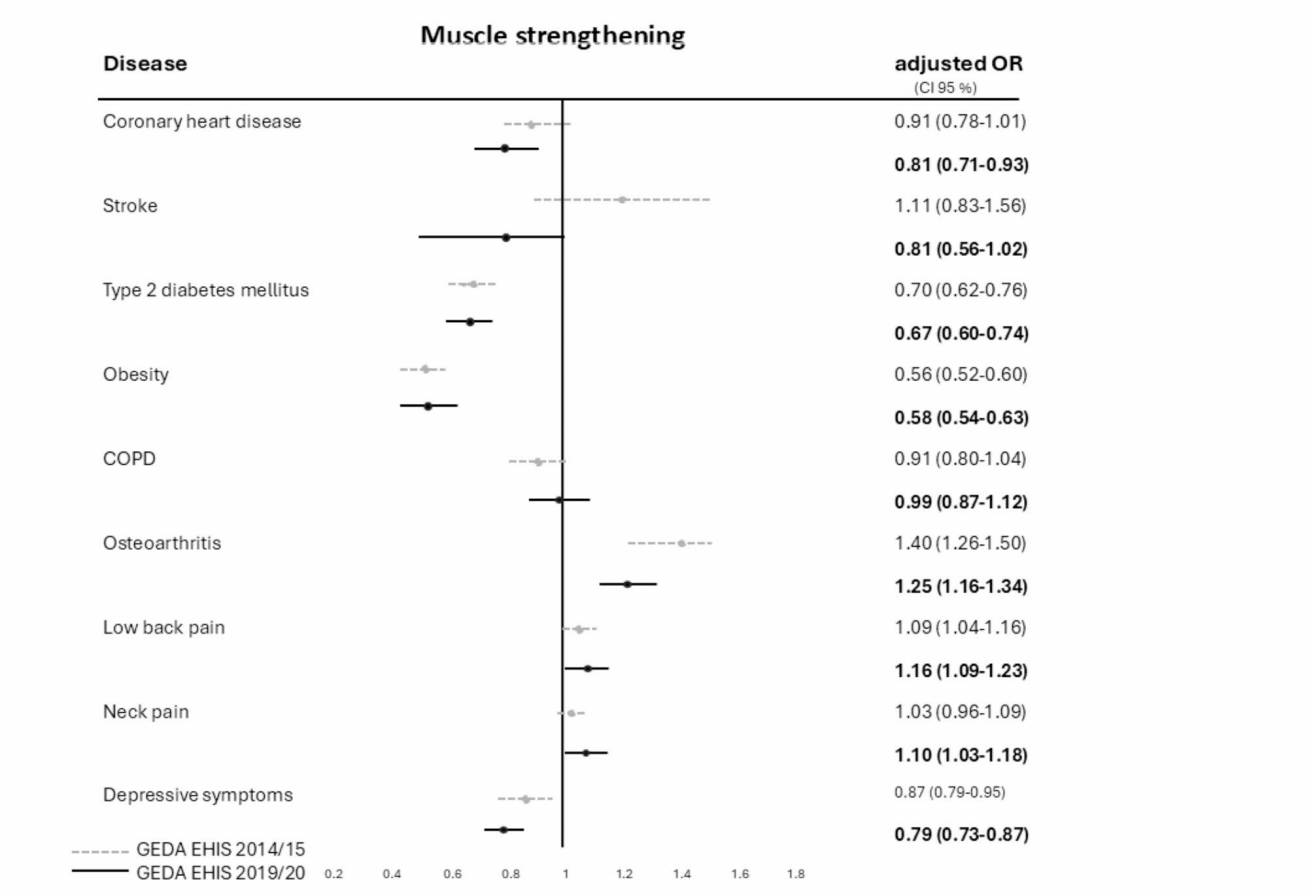
Forest plot for multivariate logistic regression with odds ratios (OR), adjusted for sex, age group and educational level and 95% confidence intervals (CI) for the fulfilment of recommendations for muscle-strengthening (at least two times per week) for nine NCD in two national health surveys (GEDA 2014/15-EHIS and GEDA 2019/20-EHIS) in Germany. Reference category: certain NCD not reported

Regarding muscle-strengthening, the association was strongest for obesity (OR = 0.58 [95% CI 0.54–0.63%]) type 2 DM (OR = 0.67 [95% CI 0.60–0.74%]), depressive symptoms (OR = 0.79 [95% CI 0.72–0.87%) and CHD (OR = 0.81 [95% CI 0.71–0.93%]). For adults reporting COPD, osteoarthritis, low back pain and neck pain the association was not significant. Positive associations can only be seen in adults with osteoarthritis with higher rates of muscle-strengthening compared to adults without this specific disease. The fulfilment of muscle-strengthening in adults with NCD showed a stronger association since 2014/2015 (compare GEDA EHIS-2014/15) and remains almost as high as the general adult population (see Fig. 3).

**Fig. 4:**
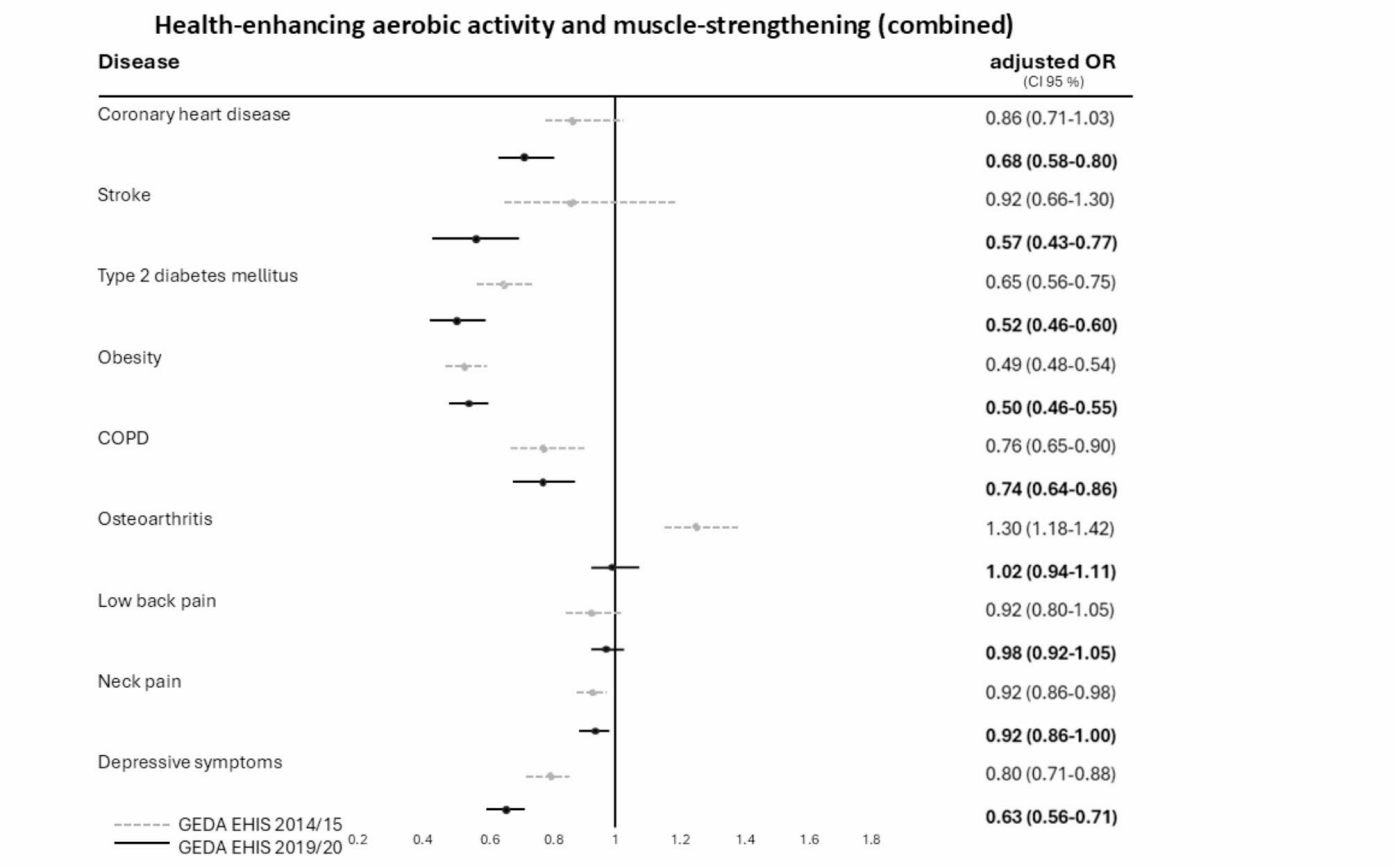
Forest plot for multivariate logistic regression with odds ratios (OR), adjusted for sex, age group and educational level and 95% confidence intervals (CI) for the fulfilment of recommendations for both WHO recommendations (health-enhancing aerobic PA and muscle-strengthening combined) for nine NCD in two national health surveys (GEDA 2014/15-EHIS and GEDA 2019/20-EHIS) in Germany. Reference category: certain NCD not reported

Figure 4 shows changes in the fulfilment of both activity recommendations combined (aerobic PA, muscle-strengthening), with meaningful reductions between 2014/15 and 2019/20 in CHD, stroke, type 2 DM and depressive symptoms and significantly lower rates of WHO PA recommendation fulfilment compared to adults without the specific NCD. The positive trend of higher activity rates in adults with osteoarthritis in 2014/15 cannot be seen any more in 2019/20. The fulfilment of both activity recommendations in adults with NCD tend to be stable since 2014/2015 (compare GEDA EHIS-2014/15) and remains low compared to the general adult population.

### Motivational readiness for PA behavior change

The stages of motivational readiness for PA behavior change of participants in GEDA 2014/15-EHIS reporting any of the specific NCD in comparison to the population without this specific NCD are described in Table 3.

The prevalences of adults in the precontemplation stage (i) (*not considering to increase PA level within 6 months)* ranged from 37.5% [95% CI 35.1–40.0%] for depressive symptoms to 58.5% [95% CI 50.6–66.5%] for stroke, while the general adult population reported a rate of 40.8% [95% CI 40.0-41.6%]. Prevalence rates of being in the contemplation stage (ii) (*considering to increase PA level within 6 months*) among adults with NCD differed from 5.4% [95% CI 2.1–8.8%] for stroke to 16.1% [95% CI 14.2–18.0%] for depression, while 13.3% [95% CI 12.7–13.8%] of the general adult population were categorized as being in this stage. For being in preparation for PA (iii) (*with the intention of increasing PA levels within 30 days*) the reported prevalence of persons with NCD ranged from 18.3% [95% CI 12.4–24.2%] for stroke to 30.0% [95% CI 28.4–32.0%] for obesity, while the general adult population reported 24.7% [95% CI 24.0-25.4%]. The prevalence rates of persons with NCD in the action stage (iv), where adults have already started to exercise more, was almost equally distributed between persons with NCD (from 0.9% [95% CI 0.6–1.3%] for osteoarthritis to 2.7% [95% CI 2.0-3.5%] for depressive symptoms), and the general adult population (2.0% [95% CI 1.8–2.3%]). In the maintenance stage (v), where the desired level of PA is sustained, prevalence rates ranged widely within persons with NCD (from 20.5% [95% CI 18.9–22.1%] for osteoarthritis to 10.3% [95% CI 9.2–11.5%] for obesity), while 19.1% [95% CI 18.5–19.7%] in the general adult population were categorized as maintainers.

**Table 3:** Motivational readiness (stages of change) for physical activity in groups with different NCD and no NCD; (GEDA 2014/15-EHIS)

	General Population		CHD		Stroke		Type 2 diabetes mellitus		Obesity		COPD		Osteoarthritis		Low back pain			Neck pain			Depressive symptoms	
Cases (%)	22,641		907 (4.0)		249 (1.2)		1,712 (7.7)		4,049 (18.1)		1,230 (5.8)		3,897 (17.9)		9,040 (40.1)			7,976 (34.3)			2,499 (11.3)	
Motivational readiness for PA (%)																						
Pre-contemplation	40.8		53.5	(49.2–57.9)	**58.5**	(50.6–66.5)	53.4	(50.5–56.6)	42.0	(40.0-43.9)	50.8	(47.2–54.4)	46.3	(44.3–48.1)	41.0	(39.7–42.3)	39.4		(38.0-40.7)	37.5		(35.1–40.0)
Contemplation	13.3		8.5	(6.1–11.0)	5.4	(2.1–8.8)	11.5	(9.5–13.4)	15.7	(14.3–17.2)	11.6	(9.3–14.9)	10.7	(9.5–11.9)	13.4	(12.5–14.3)	14.1		(13.1–15.1)	**16.1**		(14.2–18.0)
Preparation	25.0		19.4	(16.0-22.8)	18.3	(12.4–24.2)	22.4	(19.9–25.0)	**30.0**	(28.4–32.0)	22.3	(19.4–25.2)	21.6	(19.9–23.2)	25.8	(24.6–27.0)	27.2		(26.0-28.4)	29.4		(27.2–31.7)
Action	2.0		2.1	(0.8–3.5)	1.9	(0.2–3.6)	1.6	(0.7–2.4)	1.8	(1.3–2.3)	1.9	(1.0-2.8)	0.9	(0.6–1.3)	1.9	(1.6–2.8)	2.0		(1.7–2.4)	**2.7**		(2.0-3.5)
Maintenance	19.1		16.4	(13.9–19.8)	15.8	(9.6–22.1)	11.0	(9.2–12.8)	10.3	(9.2–11.5)	13.4	(10.9–15.9)	**20.5**	(18.9–22.1)	17.8	(16.9–18.8)	17.2		(16.2–18.3)	14.2		(12.5–15.9)

## Discussion

We analysed PA among adults with certain NCD for GEDA 2019/20-EHIS in comparison to the population without this specific NCD. Furthermore, we observed temporal trends of PA between the two German health monitoring surveys GEDA 2014/15-EHIS and GEDA 2019/20-EHIS. Our analysis of GEDA 2019/20-EHIS PA data showed that PA and motivational readiness for PA remains low in adults with NCD, with differences between the NCD. PA behavior of adults with NCD has been first analysed for Germany by Sudeck et al. in 2021 [14]. Our analyses are based on the same definitions and therefore our methodological approach is almost identical to those of Sudeck et al. and suitable for trend analysis. PA for health promotion is strongly recommended for adults with and without NCD in national and international PA guidelines like the Global Action Plan on Physical Activity 2018–2030 of the WHO or the German guidelines for PA [35, 39].

The analyses of GEDA 2019/20-EHIS underline that PA is low in adults with NCD, whether you focus on health-enhancing aerobic PA or muscle-strengthening. The results also show a wide range of PA prevalence for different NCD. For example, adults with musculoskeletal diseases like lower back pain, neck pain or osteoarthritis are almost as physically active as the population without this specific disease. Adults reporting COPD, CHD, obesity and depressive symptoms were found to be highly inactive in regards to health-enhancing aerobic activity while adults reporting stroke and type 2 DM were found to be particularly inactive in both dimensions. With regards to motivational readiness for PA the analysis found that the majority of adults with NCD were in the no intention group (precontemplation). At the same time, the findings show that a great amount of adults reporting NCD are ready to change PA behavior (contemplation and preparation group together ranged between 23 and 45%). Motivational readiness to increase PA was observed to be higher in adults with obesity, low back pain, neck pain and depressive symptoms compared to adults reporting CHD, stroke, type 2 DM and COPD. Our results show that little has changed regarding aerobic PA in adults with NCD since 2014/2015 in Germany, with a negative trend for adults reporting stroke and osteoarthritis. One positive trend which was identified is that muscle-strengthening has increased in almost all NCD except for adults reporting stroke. In the general adult population, both PA dimensions have significantly increased over time by 2.7% for aerobic PA and by 6.9% for muscle-strengthening in 2019/20 compared to 2014/15. However, a current study by Strain et al. reports that in 2022 only 12.0% (9.2-15.1%) of the German adult population were not sufficiently physically active [13]. One reason for the differences could be the use of a different PA questionnaire. The International Physical Activity Questionnaire short form (IPAQ-SF) was used in the Eurobarometer 2022, which was the data source for the analysis of Strain et al., includes walking, moderate-intensity activities and vigorous intensity activities, while in our analysis walking was excluded, because of not meeting the threshold for moderate PA. Furthermore, the admittedly large difference in the fulfilment of PA recommendations may be due to a different data source used by Strain et al. and a much smaller sample size (*N* = 1,466) compared to the national representative GEDA EHIS survey we used for our analyses.

Our results regarding low PA among adults with NCD are not only in line with GEDA 2014/15 but also with the results of a study by Brawner et al. (2016) which reported a low PA prevalence among adults with myocardial infarction, DM, kidney diseases, stroke and COPD compared to the general population [20]. To date, only a few surveys have looked at trends of PA behavior in adults with NCD in European countries over time and results have been diverging. While Llamas-Saez et al. in 2023 found a positive trend of PA behavior over time among adults with NCD in Spain between 2014 and 2020 [40], a study from Austria conducted by Dorner et al. 2021 found a negative trend of PA behavior in adults with DM [41]. Since PA produces multiple health benefits not only but especially for adults with NCD, countries like Germany should establish and/or expand a monitoring system on PA for adults with NCD for a better understanding and monitoring of population health as recommended by the WHO Global Action Plan on Physical Activity 2018–2030 (GAPPA) [22].

One reason why the proportion of adults with osteoarthritis, low back and neck pain who fulfil the recommendations for muscle-strengthening is higher and almost equal compared to the general adult population without this specific NCD and adults with these musculoskeletal diseases are more ready to increase or maintain PA behavior might be that the health services infrastructure is better for these conditions compared to others. For example, physicians can easily prescribe physiotherapy for persons with low back or neck pain and costs are covered by the health insurances. In the best case adults are then treated according to the guidelines with PA and muscle-strengthening exercise [42].

Our findings suggest that an adequate infrastructure in ambulatory health care, which considers motivational readiness to change behavior and includes tailored interventions to initiate regular PA and exercise as a valuable health resource is needed to address the growing number of adults living with one or more NCD. Although, there is convincing scientific evidence for the treatment with PA and exercise for most NCD, most countries, and this is also true for Germany, have no measures for sport and exercise therapy for cardiovascular, metabolic or lung diseases implemented in primary health care. Exercise specialists like sports therapists are well trained in treating patients with diseases like CHD, DM, cancer, lung diseases and musculoskeletal disorders with PA and exercise in disease specific training groups. So far, in Germany this is, however, mainly implemented in the tertiary rehabilitation settings (ambulatory and stationary) for which coverage is limited and should be expanded to additional health care facilities, including to primary health care settings. Programs should also be needs-oriented taking into account the specific needs, limitations and illnesses of individuals to ensure everyone can participate at their own pace and level. Our results also suggest that there is great potential to increase PA in NCD through targeted measures that are tailored to the different stages of motivational readiness, especially for adults in the contemplation and preparation group. In order to design tailored interventions further research and qualitative insights are, however, needed to better understand factors that influence individuals’ readiness for behavior change in adults with NCD, including reasons for low motivational readiness for persons with NCD like stroke or diabetes. Exercise therapy is safe and effective, evidence based and implemented in numerous disease specific treatment guidelines as well as in international health promotion strategies [8–10, 42–44]. Several countries already developed different approaches in ambulatory health care to promote PA in adults with NCD. Studies from Sweden (‘*Physical activity on prescription*’), England (‘*Exercise on referral*’) or New Zealand (‘*Green prescription*’) demonstrate that exercise referral schemes have advantages when interdisciplinary teams of different health care professionals are in place, with higher adherence rates to PA following PA prescription [45–47].

Our results confirm the great importance of integrating comprehensive low-threshold approaches that are tailored to the specific needs of the different NCD groups in the ambulatory health care system to promote PA among adults with NCD in Germany. Multiple inter- and multidisciplinary projects of medical and exercise professionals have been developed to promote PA in adults with different NCD, from which the health care system can draw on. For example, the *ImPuls* project is a structured transdiagnostic group-based PA intervention for patients with psychological disorders (major depressive disorders, insomnia, agoraphobia, panic disorder, or post-traumatic stress disorder (PTSD)). The project has been successfully evaluated and has recently shown that the group exercise therapy by qualified exercise professionals’ is effective in reducing global symptom severity and symptoms of depression, general anxiety, panic, and PTSD at six and 12 months assessment [48]. This approach demonstrated the potential for scaling up on a nationwide level and the integration into the German health care system. The *MoveOnko* project addresses patients with cancer by implementing a multi-professional health care structure to promote needs-oriented PA programs in combination with home-based PA treatment for oncological patients [49]. The *BewegtVersorgt* project developed and implemented a regional PA and exercise counselling and referral structure in primary health care, where physicians can prescribe PA to patients with NCD and refer them to exercise specialists [50–52]. These projects and initiatives can serve as good examples, how PA can be integrated in routine health care to promote PA in patients with NCD. Especially because these projects adopt a behavior-oriented approach and empower patients to change their behavior, effects can be expected not only at the level of PA but also at the motivational level for motivational readiness to change.

### Strength and limitations

A major strength of our analysis is the large and representative nationwide database of two GEDA surveys with combined more than 40,000 participants. Furthermore, validated questionnaires were used to assess data on PA and motivational readiness for PA [24, 34, 37]. Our findings are, however, limited as the data on PA behavior, NCD and motivational readiness are based on self-reports which may lead to over- or under-reporting due to recall bias. While the definition of variables in GEDA 2014/15-EHIS and GEDA 2019/20-EHIS is identical, changes in the survey method must be considered. While GEDA 2014/15-EHIS was based on a self-administered questionnaire, which was completed either as a paper or online questionnaire, GEDA 2019/20-EHIS is based on telephone interviews [27, 53]. The data from the GEDA 2014/15-EHIS survey is comparable with the GEDA 2019/20-EHIS as the questionnaire has remained largely unchanged. For example, GEDA 2014/15-EHIS had a list of ten NCD including cancer, but the item for cancer was not asked in GEDA 2019/20-EHIS anymore. Therefore, no PA trends for cancer could be analysed. In addition, our analysis provides for the first time the prospect of more in-depth investigations for the relationship between motivational readiness and PA of adults reporting NCD, albeit the items of motivational readiness were only available for GEDA 2014/15-EHIS and not collected in GEDA 2019/20-EHIS. Therefore the analysis on motivational readiness is restricted to a descriptive analysis and no trends over time are available.

Furthermore, for GEDA 2019/20-EHIS the survey was carried out during the COVID-19 pandemic and during the spring lockdown in 2020. Social isolation have had a negative effect on PA behavior of individuals in general [54]. Particular in adults reporting NCD who are at higher risk of severe COVID-19-related illness and death. This may have led to lower PA rates in the 2019/20 survey compared to 2024/15 and may be partly an explanation for negative activity trends in our analysis. In addition, in our results of GEDA 2014/15 EHIS it is noticeable that there are small differences to the published data of Sudeck et al. 2021 [14]. For example, the differences for the prevalence of depression symptoms. We used the self-reported 12-month prevalence for depression in both GEDA data sets, while Sudeck et al. used a binary indicator for at least moderate depressive symptoms, asking for the last two weeks based on the 8‑item version of Patient Health Questionnaire (PHQ-8). Finally, we used fewer covariates in our analyses, while Sudeck et al. included socioeconomic status as a covariate in the regression model, we used sociodemographic as covariates [14].

## Conclusion

In conclusion, our analysis is the first to compare PA over time and motivational readiness for PA change in adults with NCD in Germany. Our results show that PA as well as motivational readiness for PA vary between adults reporting the studied NCD. Thus, a differentiated look must be taken at different types of NCD and PA dimensions (health-enhancing aerobic PA, muscle-strengthening) and the differences in regards to the motivational readiness of adults with NCD when planning PA interventions. Based on our findings, new approaches to encourage adults with NCD to engage in more PA and exercise and to use existing PA programmes are urgently recommended for Germany with a particular attention to motivational readiness. Health and exercise physiologists, sports physicians, sport/exercise scientists and other exercise specialists should be integrated into the health care system to improve health care and motivate and strengthen patients’ competencies for PA. Finally, structured and interdisciplinary PA programs for patients with NCD in ambulatory care are strongly recommended and needed.

## Data Availability

We used the Scientific Use File of the German Health Update (GEDA 2014/2015-EHIS) and German Health Update (GEDA 2019/2020-EHIS). The datasets used and/or analysed are available from the RKI upon reasonable request.

## Abbreviations

CHD: Coronary heart diseases
CI: Confidence intervals
CVD: Cardiovascular disease
CRD: Chronic respiratory disease
COPD: Chronic obstructive pulmonary disease
DM: Diabetes mellitus
DMT2: Diabetes mellitus type 2
GAPPA: Global Action Plan on Physical Activity 2018–2030
GEDA: German Health Update
MVPA: Moderate to Vigorous Physical Activity
NCD: Non-communicable diseases
PA: Physical activity
PHQ-8: Patient Health Questionnaire depression scale
PTSD: Post-traumatic stress disorder
OR: Odds ratio
RKI: Robert Koch Institute
TTM: Transtheoretical model
WHO: World Health Organisation
